# What have other jurisdictions done about dementia care pathways?

**DOI:** 10.1002/alz70858_103688

**Published:** 2025-12-26

**Authors:** Larry Chambers, Saskia Sivananthan, Alexandra Whate, Kedron Raju, Alixe Ménard

**Affiliations:** ^1^ The Brainwell Institute, Toronto, ON, Canada; ^2^ McMaster University, Hamilton, ON, Canada; ^3^ McGill University, Montreal, QC, Canada; ^4^ University of Waterloo, Waterloo, ON, Canada; ^5^ University of Victoria, Victoria, BC, Canada; ^6^ University of Ottawa, Ottawa, ON, Canada

## Abstract

Dementia care pathways are important tools that help standardize care, improve patient outcomes, and support both clinicians and individuals living with dementia.

We analyzed dementia strategies in countries with healthcare systems similar to Canada's. Our research reviewed WHO member states’ dementia plans, reports from Alzheimer's Disease International, and other global databases. We focused on countries with federated health systems, similar resources, and comparable insurance models. We assessed strategies based on governance, funding, accountability, and clearly defined goals. Strong leadership and governance are essential for effective implementation of dementia care pathways. A central organization should oversee coordination while ensuring all levels of government have clear roles and responsibilities. A well‐structured implementation plan should outline key initiatives, target populations, and measurable outcomes. Adequate funding, staffing, and infrastructure are necessary to support these efforts. While long‐term financial investment is critical, having a trained workforce with the capacity to implement changes is equally important. Collaboration across different levels of government and stakeholder buy‐in are also essential to ensure success. Accurate data systems help track progress, enabling real‐time monitoring and public reporting. Regular evaluations allow for adjustments and improvements. Canada must develop a strong implementation plan to provide effective dementia care pathways. By learning from other countries, setting clear targets, and improving coordination, Canada can improve dementia care, treatment, and prevention nationwide.

